# Financial strain and depression in the U.S.: a scoping review

**DOI:** 10.1038/s41398-023-02460-z

**Published:** 2023-05-13

**Authors:** Catherine K. Ettman, Alice Y. Fan, Alexander P. Philips, Gaelen P. Adam, Grace Ringlein, Melissa A. Clark, Ira B. Wilson, Patrick M. Vivier, Sandro Galea

**Affiliations:** 1 Boston University School of Public Health, Boston, MA USA; 2 Brown University School of Public Health, Providence, RI USA; 3 Johns Hopkins Bloomberg School of Public Health, Baltimore, MD USA; 4 Tufts University School of Medicine, Boston, MA USA

**Keywords:** Depression, Human behaviour

## Abstract

While the association between assets and depression has been established, less is known about the link between financial strain and depression. Given rising financial strain and economic inequity due to the COVID-19 pandemic, understanding the role that financial strain plays in shaping population depression in the United States is particularly salient. We conducted a scoping review of the peer-reviewed literature on financial strain and depression published from inception through January 19, 2023, in Embase, Medline via PubMed, and PsycINFO, PsycArticles, SocINDEX, and EconLit via Ebsco. We searched, reviewed, and synthesized the literature on longitudinal studies on financial strain and depression conducted in the United States. Four thousand and four unique citations were screened for eligibility. Fifty-eight longitudinal, quantitative articles on adults in the United States were included in the review. Eighty-three percent of articles (*n* = 48) reported a significant, positive association between financial strain and depression. Eight articles reported mixed results, featuring non-significant associations for some sub-groups and significant associations for others, one article was unclear, and one article reported no significant association between financial strain and depression. Five articles featured interventions to reduce depressive symptoms. Effective interventions included coping mechanisms to improve one’s financial situation (e.g., mechanisms to assist in finding employment), to modify cognitive behavior (e.g., reframing mindset), and to engage support (e.g., engaging social and community support). Successful interventions were tailored to participants, were group-based (e.g., they included family members or other job seekers), and occurred over multiple sessions. While depression was defined consistently, financial strain was defined variably. Gaps in the literature included studies featuring Asian populations in the United States and interventions to reduce financial strain. There is a consistent, positive association between financial strain and depression in the United States. More research is needed to identify and test interventions that mitigate the ill effects of financial strain on population’s mental health.

## Introduction

Depression is a common mood disorder, affecting millions of Americans and their families, employers, and communities. Almost one quarter of adults in the United States report symptoms consistent with depressive disorder at some point in their lives [1]. Depression is costly to individuals and communities [2] and is associated with a number of negative health indicators including substance misuse and early mortality [3]. Depression is also highly sensitive to social and economic conditions [4–6]. As such, changes in depression in response to economic conditions may present themselves much sooner than physical health indicators and may be an effective area for intervening to prevent worsening mental and physical illness.

Social and economic factors shape population’s mental health [7, 8]. Economic indicators such as having fewer assets are associated with depression [6, 9–11]. For example, having lower income [12], having less money in savings [9, 11], and not owning a home [11] are all objective economic assets that have been associated with higher prevalence of depression in US adults. Social assets such as educational attainment and marital status are also associated with depression, with greater education and being married, each associated with less depression [10]. Beyond objective assets, perceptions of limited assets—manifesting as financial strain—may additionally [13] and even more strongly predict depression than do objective economic indicators [14, 15].

Financial strain is a correlated, but separate, construct than objective financial assets. Financial strain broadly refers to the ability of people to cover their expenses with assets available, whether measured as the perception of strain or reactions to their inability to pay for needs. In this way, financial strain is a measure of the perception of assets. Financial strain may be a function of spending proportionate to resources available and perceptions of the ability to pay for needs; thus, financial strain may be relative, shaped by the perception of others’ prosperity. For example, in a study of older adults following the Great Recession from 2007–2009, overall financial strain and depression decreased between 2006 and 2010 despite people having objectively fewer assets than before the recession [15]; people may have perceived that others around them were worse off than they were, lessening the psychological blow of having lower home values, lower income, and lower savings [15]. Thus, it is possible that social comparison may influence perceptions of or reactions to needs and relative social standing [16]. Changes in the public dialog about the overall financial status of the population may also change people’s outlook on their own financial situations [17]. Psychological strain is itself associated with poor mental health [18]. Thus, it is possible that whether or not persons feel under financial strain may matter as much as, or more than, whether they do have adequate assets [15, 17]. While improving objective economic conditions may contribute to reductions in population depression, more information is needed about how financial strain specifically is independently associated with poor mental health and how to alleviate it.

Despite a growing awareness of how adverse life events may instigate poor mental health [19–21] and how a worse socioeconomic context influences the treatment of depression [22] (affecting the likelihood of recurrence [23, 24]), few reviews have assessed the relation between financial strain and depression. Given the complex relations between access to assets, perceptions of resources, and social comparison, documenting what we know about financial strain and depression may improve our understanding of the link between the two and point to potential interventions to reduce depression. Buckman et al. reviewed studies that assessed treatment prognosis among patients with depression across a range of socioeconomic indicators that include financial strain [25]. Guan et al. reviewed observational studies on financial strain and depression globally; their assessment included cross-sectional studies and concluded with a call for more research with longitudinal data [26]. This paper contributes to the literature by focusing on studies with longitudinally collected data that were conducted in the United States.

Understanding the relation between financial strain and depression is critical, particularly as the United States emerges from the COVID-19 economic recession that has disproportionately affected low-income populations. The COVID-19 pandemic has clearly increased economic inequities in the United States, with high-income earners emerging with more and low-income earners emerging with fewer assets than before the pandemic [27]. Financial strain also likely increased following the COVID-19 pandemic, particularly among lower-income adults [28, 29]. While interventions to improve objective financial assets for people with fewer assets are critical, such efforts may be costly and politically fraught; interventions to address financial strain may be an additional lever in addressing and improving population mental health [17].

This paper aimed to scope the literature on financial strain and depression in the United States. Our research questions were: (1) What is the scope and nature of the peer-reviewed literature on financial strain and depression? (2) What is the relation between financial strain and depression? and (3) What interventions, if any, have been found to improve depression in the context of financial strain?

## Methods

We conducted a scoping review in accordance with PRISMA-ScR guidelines [30].

### Search strategy

We searched Embase, Medline via PubMed, and PsycINFO, PsycArticles, SocINDEX, and EconLit via Ebsco from inception to January 19, 2023, using keywords and terms adapted to each database. A detailed search strategy can be found in Appendix A. Citations were deduplicated in EndNote. Abstracts were then loaded and screened for inclusion in Abstrackr software (http://abstrackr.cebm.brown.edu/). Abstrackr uses machine learning to predict the likelihood of relevance for each citation [31, 32]. Titles and abstracts were screened for eligibility by the lead author (CE) and colleague (GR), and the Abstrackr software was used to prioritize relevant citations through the screening process. The full-text screening was carried out by the lead author (CE) and three additional readers (AF, AP, and GR). We also screened reference lists of related studies for relevant citations.

### Inclusion and exclusion criteria

The following inclusion and exclusion criteria were determined in accordance with the guidance for systematic scoping reviews [33, 34].

#### Types of participants

We included studies featuring adults who were 18 years or older. We excluded studies about young adults that had aggregated samples with persons younger than 18 years of age.

#### Concept

We included longitudinal, quantitative articles that assessed the concept of depression and financial strain. Articles that assessed mental health broadly but did not disaggregate results by depression or psychological distress were not eligible for inclusion. Articles that did not measure subjective perceptions of financial strain (for example, articles that reported on objective measures of financial standing, such as wealth but did not have measures of financial strain) were excluded. We excluded studies that only controlled for financial strain as a covariable in their models and did not report on the relation between depression and financial strain. We also excluded articles that focused on postpartum depression, given the unique features of that important phenomenon [35]. We included related constructs such as economic stress, strain, or financial problems.

#### Context

We included studies conducted in the United States. The aim of the study was to understand the literature on financial strain and depression in the United States, given the varying nature of economic and financial strain across contexts; by limiting to the United States, we hoped to focus on literature within one country context to better understand the evolution of the understanding of these concepts in the United States over time.

#### Types of evidence sources

Articles published in the peer-reviewed literature were eligible for inclusion. Only articles published in the English language were included.

### Data charting

We extracted the following information from eligible articles: year of publication, data source, sample size, sample description, sampling technique, study length, the definition of financial strain, number of items in the financial strain definition, number of times financial strain was measured, definition of depression, the relation between financial strain and depression, and a standardized summary. We calculated the percentage of articles by sampling technique, gender of populations studied, race/ethnicity of populations studied, number of items included in financial strain definition, number of times financial strain was measured, and assessment of depression used. We flagged studies that included and reported findings on depression-reduction interventions. We used a narrative synthesis to summarize the results.

## Results

**Fig. 1:**
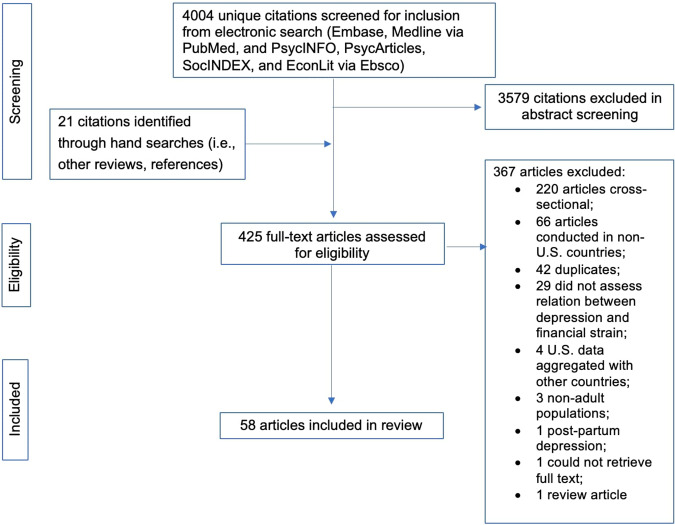
PRISMA flowchart of the study selection process.

We screened 4004 unique citations for inclusion. Four hundred twenty-five abstracts were identified and reviewed in full text for eligibility; 367 full-text articles were excluded (*n* = 220 cross-sectional; *n* = 66 conducted in non-US countries; *n* = 42 duplicates; *n* = 1 could not retrieve full text; *n* = 1 review article; *n* = 29 did not assess relation between depression and financial strain; *n* = 3 non-adult populations; *n* = 1 postpartum depression; *n* = 4 US data aggregated with other countries). In total, 58 longitudinal, quantitative studies conducted in the United States were included in the review. Figure 1 shows the PRISMA flowchart of the study selection process. Appendix B shows the alphabetized citations of the 58 articles included in the review.

### Article characteristics

Included articles were published between 1981 and 2022. Three studies were analyzed in multiple papers: the JOBS II study (*n* = 4), the Hispanic Established Population for Epidemiological Studies of the Elderly (*n* = 3), and the Iowa Youth and Family Project (n = 2). All other articles used a data source that only appeared once (*n* = 39) or was original to that study (*n* = 10). Table 1 shows the characteristics of the articles included in the review. Seventy-four percent of studies (*n* = 43) featured adults ages 18 years and older; 25.9% (*n* = 15) featured sample populations ages 50 years or older. Forty-seven percent of articles (*n* = 27) used a probability-based sampling design; 50% (*n* = 29) used a non-probability-based sampling design; 3.4% (*n* = 2) did not provide the sampling design. Seventy-two percent (*n* = 42) included male and female populations; 28% (*n* = 16) of articles featured only female populations; no articles featured only male populations. The majority of articles (74.1%, *n* = 43) featured populations identifying as White or Black (67.2%, *n* = 39). Forty-five percent of articles (*n* = 26) included “other” race/ethnicity groups, 32.8% (*n* = 19) explicitly stated that they included Hispanic persons, and 19% (*n* = 11) featured Asian persons.

**Table 1:** Characteristics of included articles (*N* = 58).

	Frequency (*n*)	Percent (%)
Total	58	
**Life stage**		
Adults (ages 18 years and over)	43	74.1
Older adults (ages 50 years and over)	15	25.9
**Gender of samples**		
Female and male	42	72.4
Female only	16	27.6
Male only	0	0.0
Racial/ethnic groups included*		
Asian, non-Hispanic	11	19.0
Black, non-Hispanic	39	67.2
Hispanic	19	32.8
White, non-Hispanic	43	74.1
Other	26	44.8
Not provided	3	5.2
**Sampling design**		
Probability based	27	46.6
Non-probability based	29	50
Not provided	2	3.4
**Financial strain definition items**		
1-item	11	19.0
2-item	7	12.1
3-item	14	24.1
4-item	6	10.3
5-item	2	3.4
6-item	5	8.6
7-item	2	3.4
9-item	1	1.7
11-item	1	1.7
26-item	1	1.7
27-item	2	3.4
32-item	1	1.7
Did not list	2	3.4
**Number of time points at which financial strain was measured**		
1	12	20.7
2	21	36.2
3	10	17.2
4	6	10.3
5	3	5.2
6	1	1.7
7	2	3.4
21	1	1.7
Not provided	1	1.7
**Assessment of depression**		
CES-D	30	51.7
Hopkins symptom checklist	10	17.2
University of Michigan Composite International Diagnostic Interview	2	3.4
PHQ-2	2	3.4
PHQ-9	2	3.4
Barber et al. 2001	1	1.7
Beck Depression Inventory	1	1.7
Brief Symptom Inventory	1	1.7
CIDI-SF	1	1.7
Depression anxiety stress scale	1	1.7
Diagnostic and Statistical Manual of Mental Disorders, Fourth Edition	1	1.7
Geriatric depression scale	1	1,7
Hamilton depression rating scale	1	1.7
ICD-10 Diagnosis code	1	1.7
K6	1	1.7
Latent factor of psychological distress from DASS-21 and CES-D-20	1	1.7
Original	1	1.7

^*^Does not add up to 58 since studies could feature more than one race/ethnicity group. Two studies did not list the racial/ethnic groups studied.

*CES-D* Center for Epidemiological Studies—Depression, *PHQ-9* Patient Health Questionnaire-9, *K6* Kessler-6, *CIDI-SF* World Health Organization’s Composite International Diagnostic Interview—Shortform.

### Definitions of financial strain and depression

Financial strain was defined in a variety of ways. Almost three-quarters of the papers studied (74.1%; *n* = 43) used a measure for financial strain that included one, two, or three items. The earliest definition of financial strain that we included in the review was the 27-item Peri Life Events Scale by Dohrenwend et al. (1978), including the following indicators: “borrowed money to help pay bills,” “sold possessions or cashed in life insurance,” “changed food shopping or eating habits to save money,” and “sold the property to raise money” among others [36]. A more commonly used definition of financial strain emerged in 1981: a 9-item scale created by Pearlin et al. [37]. The scale included items ranging from the ability to pay for clothing, medical care, housing, food, leisure activities, transportation, and furniture; difficulty paying bills overall; and the ability to “make ends meet.” Later studies used different combinations of these nine items depending on the focus of their work. Six studies, published from 1987 through 2010, used some combination of the original Pearlin definition. Three studies used financial strain definitions derived from Conger et al. (1994), which assessed the ability of parents to make ends meet, to pay for what they needed “for a home, clothing, household items, a car, food, medical care, and recreational activities” and economic adjustments, or changes made in response to economic stress over the past year [38].

The remaining studies used a combination of de novo questions to define financial strain based on their specific research question. The modal number of times that financial strain was measured (as reported in the published articles) was two times (*n* = 21, 36.2%). Twenty-one percent of studies (*n* = 12) measured financial strain once and 17% (*n* = 10) of studies measured financial strain three times.

Depression was defined relatively consistently, using validated measures in most studies. The most used depression assessment was the Center for Epidemiological Studies-Depression (CES-D), reported in 51.7% of articles (*n* = 30). The next most used depression assessments were the Hopkins Symptom Checklist (17.2%, *n* = 10) and the University of Michigan Composite International Diagnostic Interview, PHQ-2, and PHQ-9 (each 3.4%, *n* = 2).

### Association between financial strain and depression

A preponderance of articles (82.8%, *n* = 48) reported a significant positive association between financial strain and depression. Eight studies reported mixed findings, either showing significance for some groups but not others (such as in men but not women) or did not show a significant association in some groups (potentially due to sample size limitations). One study reported no association between financial strain or unemployment due to the COVID-19 pandemic and trajectories of depression over the COVID-19 pandemic for parents of children with chronic pain [39]. Hertz-Palmor et al. found that adults in the United States had an association between financial strain and depression at *p* = 0.07 [28]. McCormick et al. found that high but not moderate financial strain was significantly associated with depression [40]. Gutierrez et al. found that chronic financial strain was significantly associated with depression but not acute financial strain [41]. Yoshikawa et al. found that economic hardship was significantly associated with psychological distress at 14 months but not at 24 months of follow-up [42]. Mendes de Leon et al. found that financial strain was associated with future depression in men but not women [43]. Valentino et al. found that although low depression scores predicted lower levels of financial strain, higher depression scores did not predict the severity of financial strain [44]. One study, Robinson et al., showed inconsistent findings within the paper itself (with their table and figure showing conflicting numbers) [45]. There were no studies that showed a protective effect of financial strain on depression. It is possible that the direction of the relation between financial strain and depression goes both ways: Curran et al. found that financial strain at T1 did not predict depressive symptoms at T2 for either mothers or fathers, but depressive symptoms at T1 was associated with financial strain at T2, for mothers but not fathers [46]. However, Jones et al. found that depressive symptoms did not predict later financial strain among patients with cancer [47]. Table 2 shows the charted review, describing summary details of each included article.

**Table 2:** Charted data (*n* = 58).

Author last name and year published	Data source (sampling design; study length)	Sample description and size	Financial strain definition	Depression assessment	Association between financial strain and depression	Standardized summary
Pearlin et al. 1981	Original (Probability based; 4 years)	Adults aged 18–65 in Chicago, IL. *N* = 2300	9-item scale assessing ability to afford adequate housing, appliances, a car, food, medical attention, clothing, monthly payments, and recreational activities	10-item scale assessing common depressive symptoms, such as hopelessness, lack of enthusiasm, absence of pleasure, and sleep quality	Positive	Financial strain was positively associated with depressive symptoms in the general adult population. Financial strain also mediated the relationship between job disruption and depression.
Krause 1987	City directory in Galveston, TX (Probability based; 1.5 years)	Community sample of elderly adults 65 years and older in Galveston, TX. *N* = 351	4-item scale assessing the ability to afford medical bills, clothes, food, and recreational activities (Pearlin et al., 1981)	20-item CES-D	Positive	Financial strain was positively associated with depressive symptoms in elderly persons. Locus of control also mediated the relationship between financial strain and depression.
Mendes de Leon et al. 1994	Yale Health and Aging Project (Probability based; 3 years)	Community sample of elderly adults 65 years and older in New Haven, CT. *N* = 2812	4-item scale assessing the ability to afford medical treatment, clothes, food, and recreational activities (Pearlin et al., 1981)	20-item CES-D	Mixed	Financial strain was positively associated with depressive symptoms in men. In women, no significant association was found.
Lipton 1994	Los Angeles Epidemiological Catchment Area study (Probability based; 1 year)	Non-Hispanic white adults in Los Angeles, CA. *N* = 1149	5-item scale assessing the ability to afford monthly payments, medical bills, furnishings, clothing, and food	20-item CES-D	Positive	Financial strain was positively associated with depressive symptoms in non-Hispanic white adults.
Vinokur et al. 1996	JOBS II study (Probability based; 6 months)	Recently unemployed, job-seeking adults in southeast MI. *N* = 815	3-item scale assessing the quality of life, ability to afford medical care, shelter, or food, and overall financial hardship for a living (Vinokur & Caplan, 1987)	11-item Hopkins Symptom Checklist	Positive	Financial strain was positively associated with depressive symptoms in persons seeking employment and their partners.
Vinokur and Schul 1997	JOBS II study (Probability based; 6 months)	Recently unemployed, job-seeking adults in southeast MI. *N* = 1801	3-item scale assessing the quality of life and anticipated hardship based on household income	11-item Hopkins Symptom Checklist	Positive	Financial strain was positively associated with depressive symptoms in recently unemployed, job-seeking adults. Financial strain also mediated the effect of re-employment on decreasing depressive symptoms.
Horwitz et al. 1998	Rutgers Health and Development Project (Probability based; 10 years)	Young, married individuals 25-31 years old in NJ. *N* = 458	6-item scale assessing ability to afford medical bills, furnishings, unexpected payments, cars, nourishment, clothing, and recreational activities	10-item Hopkins Symptom Checklist	Positive	Financial strain was positively associated with depressive symptoms in young married persons.
Krause and Thompson 1998	Americans’ Changing Lives Survey (Probability-based; 3 years)	Individuals aged 60 or older living in the coterminous United States (*N* = 1669)	3 items assessing financial strain based on how difficult it is to pay bills, how finances work out at the end of the month, and satisfaction with the present financial situation	6-item CES-D	Positive	Financial strain was positively associated with depressive symptoms in elderly persons. Elderly persons with cognitive impairment reported stronger associations between financial strain and depressive symptoms.
Price et al. 2002	JOBS II study (Probability based; 2 years)	Recently unemployed, job-seeking adults in southeast MI. *N* = 756	3-item scale assessing the quality of life and anticipated hardship based on household income	11-item Hopkins Symptom Checklist (Derogatis et al., 1974)	Positive	Financial strain was positively associated with depressive symptoms in persons seeking employment.
Strawbridge et al. 2002	Alameda County Study (Probability based; 5 years)	Older adults aged 50-94 years in Alameda County, CA. *N* = 1947	5-item scale assessing ability to afford clothing, medical bills, housing, and/or food (in previous 30 days)	12-item Diagnostic and Statistical Manual of Mental Disorders, Fourth Edition	Positive	Financial strain was positively associated with depressive symptoms in older adults. Exercise was protective of depression when controlling for financial strain and other demographic characteristics.
Vinokur and Schul 2002	JOBS II study and Couples Employment Project (Purposive; 2 years)	Recently unemployed, job-seeking adults in southeast MI and MD. *N* = 2243	3-item scale assessing the quality of life, ability to afford medical care, shelter, or food, and overall financial hardship for a living (Vinokur & Caplan, 1987)	11-item Hopkins Symptom Checklist	Positive	Financial strain was positively associated with depressive symptoms in persons seeking employment.
Angel et al. 2003	Hispanic Established Population for Epidemiological Studies of the Elderly (Not provided; 8 years)	Elderly Mexican-origin individuals 64 years and older in AZ, CA, CO, NM, and TX. *N* = 3050	2-item scale in an interview, assessing the ability to afford monthly bills and monthly savings (Pearlin, 1981)	20-item CES-D	Positive	Financial strain was positively associated with depressive symptoms in elderly Mexican-origin individuals.
Seto et al. 2005	Maternal Health Practices and Child Development Project (Purposive; 10 years)	Low-income mothers 18 and older. *N* = 476	3-item scale assessing the ability to afford childcare necessities and monthly payments	20-item CES-D	Positive	Financial strain was positively associated with depressive symptoms in low-income mothers. Mothers with “chronic severe” depression had the highest levels of financial strain, while mothers with “chronic moderate” depression mothers also had a statistically significant higher levels of financial strain in comparison to the mothers who once had depression or never had it.
Horwitz et al. 2007	Birth records at the State of Connecticut Department of Public Health (Probability based; 1 year)	Mothers who gave birth to a healthy child between July 1995 and September 1997 at the Yale-New Haven Hospital in New Haven, CT. *N* = 1095	1-item scale assessing financial hardship paying bills	20-item CES-D	Positive	Financial strain was positively associated with depressive symptoms cross-sectionally and over time in mothers of young children.
Kasen et al. 2008	Children in the Community study (Probability based; 30 years)	Mothers with one or more children between 1 - 10 years old living with their biological mother between the ages of 19–44 in upstate NY. *N* = 544	2-item scale assessing perception of financial status	9-item Hopkins Symptom Checklist	Positive	Financial strain was positively associated with depressive symptoms in women with a BMI greater than or equal to 30.
Jackson et al. 2008	Office of Employment Services of the New York City Human Resources Administration (Probability based; 3 years)	Single Black mothers who have received welfare assistance and had a 3- or 4-year-old child in NYC, NY. *N* = 178	2-item scale assessing the ability to afford monthly expenses and how often they chose not to purchase something because they could not afford the cost (McLoyd et al., 1994)	20-item CES-D	Positive	Financial strain was positively associated with depressive symptoms in single mothers of 3- to 4-year-old children.
Yoshikawa et al. 2008	Early Childhood Cohort of the Center for Research on Culture, Development and Education (Probability based; 2 years)	Minority adult mothers aged 18 or older in postpartum wards in 3 large New York City hospitals known to treat low-income patients had just given birth to a “healthy, full-term infant” within the past 2 days, identified as Mexican, Dominican, or African American, and live in NYC, NY. *N* = 324	4-item scale assessing if the respondents’ household has had an absence of a home phone and the ability to afford housing and other monthly bills	6-item Kessler Psychological Distress Scale	Mixed	Financial strain was positively associated with depressive symptoms at 14 months in low-income single mothers. It was not significantly associated with depressive symptoms at 24 months. Access to institutional resources predicted less financial strain, which in turn predicted fewer depressive symptoms.
Reisinger and Dilorio 2009	Project Epilepsy Awareness, Support, and Education (EASE) (Purposive; 6 months)	Persons diagnosed with epilepsy over a year prior to the start of the study who were 18-75 years old in Boston, MA and Atlanta, GA. *N* = 319	Scale assessing “patient satisfaction relating to financial aspects”	20-item CES-D	Positive	Negative attitudes towards the “financial aspects of patient satisfaction” was positively associated with depressive symptoms in persons diagnosed with epilepsy.
Krause 2009	Not provided (Probability based; 15 years)	Retired elderly adults 65 years or older. *N* = 818	3-item scale (Pearlin et al., 1981)	4-item CES-D	Positive	Financial strain was positively associated with depressive symptoms in older, retired adults. Stronger senses of gratitude, which was associated with frequent attendance in religious gatherings and stronger “God-mediated control beliefs”, weakened this relationship.
Cohen et al. 2010	Original (Purposive; 2.5 years)	Older adults 55 years or older who were diagnosed with any depressive disorder. *N* = 143	3-item scale (Pearlin et al., 1981)	20-item CES-D	Positive	Financial strain was positively associated with depressive symptoms in older adults with pre-existing depression diagnoses.
Galatzer-Levy and Bonanno 2012	Changing Lives of Older Couples (Purposive; 4 years)	Married spouses of adults 65 years or older who have died in Detroit, MI. *N* = 301	3-item scale (Pearlin et al., 1981)	9-item CES-D	Positive	Financial strain was positively associated with depressive symptoms in groups of all levels of depression immediately following the death of a partner.
Wadsworth et al. 2013	Original (Purposive; 2 years)	Low-income families with 2 parents caring for a child between the ages of 1-18 for at least 6 months in Denver, CO. *N* = 275	11-item Economic Hardship Questionnaire assessing adjustments participants have taken in order to pay bills (Lempers et al., 1989)	20-item CES-D	Positive	Financial strain was positively associated with depressive symptoms in both mothers and fathers.
Gilman et al. 2013	Prevention of Suicide in Primary Care Elderly: Collaborative Trial (Purposive; 2 years)	Primary care patients 60 years or older scored at or above 18 on the Mini-Mental State Examination in 20 primary care facilities in New York City, NY, Philadelphia, PA, and Pittsburgh, PA. *N* = 1226	1-item scale assessing ability to “make ends meet”	Hamilton Depression Rating Scale	Positive	Financial strain was positively associated with depressive symptoms in older primary care patients. Reporting financial strain and less than $200,000 in income was positively associated with even greater depressive symptoms.
Uebelacker et al. 2013	Women’s Health Initiative Observational Study (Purposive; 3 years)	Women aged 50–79 in post-menopause. N = 91912	1-item scale assessing the prevalence of “major problems with money”	6-item CES-D	Positive	Financial strain was positively associated with depressive symptoms cross-sectionally and over time in older women. Exercise did not mediate the relationship.
Newland et al. 2013	Family Life Project (Probability based; 3 years)	Families in high rural poverty in central PA and eastern NC. *N* = 1142	6-item scale assessing the ability to “make ends meet” and ability to afford housing, medical bills, clothing, and food	18-item Brief Symptom Inventory	Positive	Financial strain was positively associated with depressive symptoms in families with high rural poverty.
Szanton et al. 2014	Beat the Blues (Purposive; 4 months)	African Americans 55 years or older at the Philadelphia Senior Center, twice scored at or above a 5 on the Patience Health Questionnaire PHQ-9 2 weeks apart, and scored at or above a 24 on the Mini-Mental Status Examination in Philadelphia, PA. *N* = 208	1-item scale assessing the ability to afford life necessities	9-item PHQ-9	Positive	Financial strain was positively associated with depressive symptoms in older Black adults with pre-existing depressive symptoms. The “Beat the Blues” intervention weakened the relationship.
Valentino et al. 2014	Fast Track Project (Probability based; 6 years)	Black and white mothers with children in 2nd grade at baseline from Durham, NC, Nashville, TN, Seattle, WA, and rural PA. *N* = 668	2-item scale assessing ability to afford monthly bills and overall difficulty paying bills	20-item CES-D	Mixed	Financial strain was positively associated with depressive symptoms in Black and white mothers. Depressive symptoms did not distinguish between moderate or high levels of financial strain.
O’Neal et al. 2015	A Study on African American Marriage and Health (Probability based; 1 year)	Black heterosexual married couples living in their first year of marriage in the southeastern US. *N* = 506	6-item scale assessing the ability to afford regular payments and life necessities (Conger and Elder, 1994)	14-item CES-D	Positive	Financial strain was positively associated with depressive symptoms in recently married Black couples.
Neppl et al. 2016	Family Transitions Project (Probability based; 8 years)	White 7th graders, their parents, and a sibling within 4 years of the 7th grader in rural IA. *N* = 273	3-item scale assessing ability to afford life necessities, “can’t make ends meet”, and cutbacks participants have made because of their financial health	13-item Hopkins Symptom Checklist	Positive	Financial strain was positively associated with “parent emotional distress”, including depression.
Nam 2016	Resources for Enhancing Alzheimer’s Caregiver Health (Purposive; 1.5 years)	Dementia caregivers in Miami, FL, Boston, MA, Memphis, TN, Birmingham, AL, Palo Alto, CA, and Philadelphia, PA. *N* = 659	1-item scale assessing ability to afford rent/mortgage, medical bills, heating, and food	20-item CES-D	Positive	Financial strain was positively associated with depressive symptoms in dementia caregivers.
Wilkinson 2016	Health and Retirement Study (Probability based; 4 years)	Adults 51 years and older (US). *N* = 5366	2-item scale assessing ability to meet bills month by month and “satisfaction with one’s present financial situation”	8-item CES-D	Positive	Financial strain was positively associated with depressive symptoms in older adults.
Robinson et al. 2016	Original (Purposive; 1.5 years)	Primary unpaid caretakers who assisted dementia patients at least 4 h a week. *N* = 127	1-item scale assessing if participants were “facing financial strain”	3-item Geriatric Depression Scale	Unclear	Financial strain was positively associated with depressive symptoms in unpaid dementia caregivers. The individualized, stress-reducing intervention weakened the relationship.
Monserud and Markides 2017	Hispanic Established Population for Epidemiological Studies of the Elderly (Probability based; 18 years)	Older Mexican American adults 65 and older in TX, NM, CO, AZ, and CA. *N* = 385	1-item scale assessing the ability to afford regular payments	20-item CES-D	Positive	Financial strain was positively associated with depressive symptoms in older Mexican American adults. After controlling for church attendance and social support, there was a less but still significant relationship. After controlling for financial strain, there was no significant intercept for the recently widowed with depression.
Mitchell and Christian 2017	Original (Purposive; 30 days)	Women who were pregnant between 5–31 weeks gestation at baseline in Columbus, OH. *N* = 138	3-item scale assessing current difficulty living on household income, ability to afford rent/mortgage, medical bills, and food in the net 2 months, and quality of living (Kessler et al. 1998; Vinokur and Caplan 1987)	20-item CES-D	Positive	Financial strain was positively associated with depressive symptoms in pregnant women. Depressive symptoms acted as a mediator in the relationship between financial strain and birth weight.
Gutierrez et al. 2017	SoulPulse study (Probability based; 14 days)	Adults 19 years and older. *N* = 439	2-item scale assessing ability to afford regular bills and frequency of “financial problems”	7-item Depression Anxiety Stress Scale	Mixed	Chronic and acute financial strain was positively associated with depressive symptoms in the general adult population. In contrast, the acute financial strain was not directly associated with depressive symptoms, but it was indirectly associated with depression through “divine religious struggle”. Chronic financial strain was not indirectly associated with depression through “divine religious struggle”.
Wickrama et al. 2018	Iowa Midlife Transitions Project (Not provided; 10 years)	White European American parents and their children in rural IA. *N* = 370	27-item scale assessing specific financial hardships, such as reliance on assistance or borrowing to make necessary payments (Dohrenwend et al, 1978)	9-item Hopkins Symptom Checklist	Positive	Financial strain was positively associated with depressive symptoms in white, rural populations. It was also indirectly associated with depression through “martial hostility”.
Russell et al. 2018	Family and Community Health Study (Probability based; 11 years)	African American children 10-12 years old and their primary caretakers living in communities with a significant African American population in IA and GA. *N* = 499	32-item scale assessing the ability to afford necessities, overall feeling on the financial situation, and cutbacks participants have made because of their financial health (Conger and Elder, 1994)	University of Michigan Composite International Diagnostic Interview	Positive	Financial strain was positively associated with depressive symptoms in primary caregivers of Black children.
Buckingham-Howes et al. 2018	Original (Probability based; 4.5 years)	Adults over the age of 21 who were involved in the general seafood or tourism sectors during the Deepwater Horizon Oil Spill and lived near the Gulf Coast. *N* = 198	26-item scale assessing cutbacks participants have made because of their financial health	21-item Beck Depression Inventory	Positive	Financial strain was positively associated with depressive symptoms in persons near the Deepwater Horizon Oil Spill and were involved in the seafood or tourism industries.
McCormick et al. 2018	Lupus Outcomes Study (Purposive; 5 years)	Women diagnosed with systemic lupus erythematosus. *N* = 890	3-item scale assessing anticipated hardship living on household income and cutbacks participants have made because of their financial health	20-item CES-D	Mixed	“High” financial strain was positively associated with new-onset depressive symptoms in women with systemic lupus erythematosus. “Moderate” financial strain was positively but non-significantly associated with depression.
Barton et al. 2018	Protecting Strong African American Families program (Probability based; 17 months)	African American couples with high poverty and unemployment rates in rural GA. *N* = 346	2-item scale assessing the ability to pay bills and extra money leftover at the end of each month	20-item CES-D	Positive	Financial strain was positively associated with depressive symptoms in Black couples living in high-poverty areas.
Monserud 2019	Hispanic Established Population for Epidemiological Studies of the Elderly (Probability based; 18 years)	Older Mexican American adults 65 and older in TX, NM, CO, AZ, and CA. *N* = 1452	1-item scale assessing the ability to afford regular payments	20-item CES-D	Positive	Financial strain was positively associated with depressive symptoms in older Mexican-origin adults.
Johnson et al. 2019	Rochester Intergenerational Study (Probability based; Ongoing)	Mothers and their 8-year-old children at baseline in Rochester, NY. *N* = 385	7-item scale assessing the ability to afford rent/mortgage, food, and other life necessities	19-item CES-D	Positive	Financial strain was positively associated with depressive symptoms in women with early adolescent children.
Shippee et al. 2019	National Longitudinal Survey of Mature Women (Probability based; 36 years)	Middle-aged women, U.S. *N* = 3289	1-item scale assessing the ability to live based on household income	7-item CES-D	Positive	Financial strain was positively associated with depressive symptoms in middle-aged women. Age discrimination was indirectly associated with depression through financial strain.
Forbes and Krueger 2019	Midlife in the United States (MIDUS) (Probability based; 10 years)	Adults between ages 25–75, U.S. *N* = 3293	7-item scale assessing resulting financial burdens from the Great Recession, including paying off loans, regular payments, cutbacks, and exhaustion of unemployment benefits”	University of Michigan Composite International Diagnostic Interview—Short Form	Positive	Financial strain during the Great Recession was positively associated with depressive symptoms after the Great Recession in the general adult population.
Jones et al. 2020	National Health and Aging Trends Study (NHATS; 2011, 2012, 2015, 2016) (Purposive; 1 year)	Medicare beneficiaries aged 65+, with a diagnosis of cancer (other than nonmelanoma skin cancer) (*N* = 307)	6-item original scale assessing financial burden: problems paying off monthly credit card balances, paying bills for medical expenses over time, needing financial help from friends or family, using SNAP, needing other food assistance, and receiving help paying for utilities.	2-item PHQ-2	Positive	Financial strain was positively associated with depressive symptoms in older adults with cancer. Depressive symptoms did not predict future financial burden.
Saasa et al. 2020	Fragile Families and Child Well-being Study (Purposive; 2 years)	First-generation immigrant mothers of children aged 1 at baseline, in 20 cities across the US (*N* = 831)	12-item scale from the Basic Needs–Ability to Meet Expenses assessing financial hardship over the past year	Composite International Diagnostic Interview-Short Form (CIDI-short form)	Positive	Financial strain at Y1 was associated with depressive symptoms at Y3 in immigrant mothers of 1-year-old children. Financial strain mediated the relationship between depression and child externalizing behavior.
Bialowolski et al. 2021	Well-being survey for employees of a large US company (Probability-based; 2 years)	White-collar employees of a large US-based company (*N* = 2364)	4-item scale assessing financial safety, 1-item scale assessing financial capability, and 3-item scale assessing financial distress	ICD-10 Clinical Diagnosis of Depression from insurance claims data	Positive	Financial strain was positively associated with depression diagnosis in insurance claims data for white-collar employees at a large company.
Cao et al. 2021	APLUS: Arizona pathways to life success for university students (Purposive; 8 years)	College students at a public university in the Southwestern US, followed through their twenties (*N* = 2098)	3-item scale (Serido et al., 2010) assessing financial stress, including constantly worrying about money, having difficulty paying for things, and not feeling satisfied with financial status	4-item scale from Barber et al., 2001	Positive	Financial strain was positively associated with depressive symptoms in emerging adults.
Curran et al. 2021	Building Strong Families (Purposive; 21 months)	Lower-income individuals and their (unmarried) partners, expecting a first child (*N* = 4424)	3-item scale from Wood et al., 2010 assessing difficulty paying bills over the past year. Items included not paying the full amount of mortgage or rent; having utilities shut off due to lack of payment; and eviction.	12-item CES-D	Mixed**	Financial strain was not associated with depressive symptoms at T2 for mothers and fathers. However, depressive symptoms at T1 associated with financial strain at T2 for mothers, but not fathers.
Hertz-Palmor et al. 2021	Original (“Participants were ascertained through a crowdsourcing website (https://covid19resilience.org/) that collected data on COVID-19- related stress (worries), resilience, and mental health (Barzilay et al., 2020).”) (Purposive; 3 months)	US citizens obtained from a crowdsourcing website (*N* = 1766)	1-item original scale assessing financial burden due to COVID-19	2-item PHQ-2	Positive*	Financial strain due to the COVID-19 pandemic was not associated with depressive symptoms in the general adult population.
Law et al. 2021	Original (Parents of patients in multiple medical settings in the US) (Purposive; 4 months)	Parents of youth with chronic pain or chronic headaches across the US (*N* = 171)	1-item scale indicating experience of financial problems or unemployment due to the COVID-19 pandemic, measured at each of 4 waves and classified as “no,” “low,” or “high” economic stress	9-item PHQ-9	None***	Financial strain due to the COVID-19 pandemic did not have a statistically significant association with depressive symptom trajectories over time.
Wickrama and O’Neal, 2021	Iowa Youth and Family Project; Midlife Transitions Project; Later Adulthood Study (Purposive; 26 years)	Married, white couples with children in north-central Iowa (*N* = 254 couples)	27-item scale assessing economic difficulties experienced over the past year; created a latent construct of family financial stress based on husband-and-wife average responses to financial strain questions in 1991, 1994, and 2001	13-item SCL-R-90	Mixed	Financial strain was associated with depressive symptoms in later life for husbands but not for wives.
Adesogan et al. 2022	Protecting Strong African American Families (ProSAAF) Project (Purposive; 5 years)	Black men and women co-parenting a 9–14-year-old child (*N* = 569)	11-item scale from the Financial Adjustment Scale and 4 items from the Unmet Material Needs Scale (Conger et al.’s (1994)). The financial adjustment scale included forgoing medical insurance due to finances. The unmet material needs scale included the perception of the ability to meet family needs; the scale included having enough money to buy “the kind of home we need”.	10-item CES-D	Positive	Financial strain was positively associated with depressive symptoms in Black parents.
Galtieri et al. 2022	Larger study described by Katz et al. 2018: Trajectories of child and caregiver psychological adjustment in families of children with cancer (Purposive; 1 year)	Family members of children with cancer who serve as primary caregivers (*N* = 159)	10-item scale adapted from Barrera et al. (2001) to assess perceived financial strain (PFS). Participants asked the level of strain experienced in the past month on scales for worry “you will have to do without the basic things your family needs”, “difficulty paying bills”, not enough money for leisure and recreational activities, and changes in eating or food shopping habits to save money.	10-item CES-D	Positive	Financial strain was positively associated with depressive symptoms for family members/primary caregivers of children with cancer.
Nishtala et al. 2022	Original (Level 1 trauma center patients in Memphis, Tennessee admitted for non-neurologic injury) (Purposive; 1 year)	Recently admitted patients to a trauma center in Tennessee immediately following neurological injury (*N* = 500)	4-item scale assessing financial hardship defined by becoming unemployed, changing jobs, reporting financial problems, or having a decrease in income	CES-D (number of items not specified)	Positive	Financial strain was positively associated with depressive symptoms in the year following neurological injury for patients admitted to a trauma center.
Shelleby et al. 2022	Original (Amazon MTurk users in the US) (Purposive; 2–3 weeks)	Female primary caregivers to a child in kindergarten through 6th grade (*N* = 308)	6-item original scale assessing financial stress since the COVID-19 pandemic started: finances, job loss, eviction, losing health insurance, problems paying for medical bills, and going into debt/more debt.	The latent factor of psychological distress constructed from DASS-21 subscales and CES-D-20	Positive	Financial strain was positively associated with psychological distress in female primary caregivers of children in kindergarten.
West et al. 2022	Pathways to Health/ Caminos al Bienestar [“Caminos”] (Probability-based; 18 months)	Latina mothers of adolescents, living in Georgia (*N* = 271)	3-item scale of the Financial Strain Index (Ronald et al., 2011) measuring difficulty over the past year paying bills and essentials like food, clothing, and housing	20-item CES-D	Positive	Financial strain was positively associated with increasing trajectories of depressive symptoms over time in Latina mothers.
Wickrama et al. 2022	Iowa Youth and Family Project; Midlife Transitions Project; Later Adulthood Study (Purposive; 24 years)	Married, white women with children in north-central Iowa (*N* = 244)	4-item scale from Conger and Elder (1994) assessing family financial strain, including responses on the ability to afford “the kind of clothing we need” and “the kind of medical care we need”.	13-item SCL-R-90	Mixed	Financial strain was correlated with depressive symptoms at each time point, but not with the trajectory of depressive symptoms in married, white women. Financial strain was associated with the combined trajectory of pain and depressive symptoms. Marital closeness reduced the association of family financial strain on pain-depressive symptoms.

Note: * *p* value = 0.07. **Association was significant in the direction of depression predicting future financial strain. ***Construct of economic stress defined by unemployment or financial difficulties.

### Interventions

Five of the fifty-eight longitudinal articles featured depression reduction interventions. While the interventions did not relate directly to financial strain, some helped reduce financial strain indirectly, as detailed below. The five studies are detailed and described in chronological order.

#### JOBS II intervention for unemployed job seekers

Vinokur and Schul (1997) studied the JOBS II intervention for unemployed job seekers [48]. While three other articles used the JOBS II data, their study designs did not compare intervention arms to control groups (rather, they looked at specific arms or used study designs that did not incorporate intervention) [49–51]. The JOBS II intervention included five 4-hour sessions over 1 week for groups of 12 to 22 recently unemployed people looking for jobs. Two trainers (one man and one woman) led interactive workshops on enhancing job-search skills. The workshop taught job seekers to anticipate setbacks and overcome barriers, build self-esteem, improve job-search efficacy, and increase locus of control. The intervention was associated with re-employment, and a sense of mastery, both of which were associated with reductions in financial strain at 2- and 6-months post-intervention. Reduced financial strain was, in turn, associated with reduced depressive symptoms.

#### Fatherhood relationship and marriage education (FRAME) intervention for low-income families

Wadsworth et al. (2013) studied the Fatherhood Relationship And Marriage Education (FRAME) intervention in low-income families. FRAME was a “father-friendly, father-inclusive, family strengthening intervention” for couples [52] with three components: (1) education about strengthening marital relationships through positive communication skills, (2) stress and coping skills training, and (3) child-centered parent training. The stress and coping skill training focused on identifying life stressors such as financial strain and how to identify differences between circumstances and events that were readily solvable and those requiring different ways of coping. Couples were taught active problem-solving skills for problems with identifiable solutions (primary coping mechanisms). For stressors that could not be solved readily, couples were taught to use support, acceptance, and cognitive restructuring to cope (secondary coping mechanisms). The authors found that decreased financial strain predicted reductions in depressive symptoms for mothers and fathers. They also found that reductions in financial strain improved father-child relations.

#### Beat the blues (BTB) intervention for African American older adults

Szanton et al. (2014) studied the Beat the blues (BTB) intervention, conducted in participant homes by a licensed social worker that focused on education about depression, management of care, referral and linkage, stress reduction, and behavioral activation [53]. The population included African American adults ages 55 years or older with depressive symptoms (PHQ-9 score of 5 or greater). Participants were randomized into the BTB group (intervention group) or put on the waitlist (control group). The intervention included ten one-hour sessions weekly and then bi-weekly over a 4-month period. Components were tailored to participant care needs, preferred techniques for stress reduction, and personal activity goals. Financial strain and depression were measured at baseline and after the 4-month intervention. Participants in the BTB intervention reported lower depressive symptoms scores after the 4-month intervention relative to the control group. Participants who reported financial strain at the 4-month mark reported a 6.4-point reduction in the PHQ-9 score, while participants who reported no financial strain at the 4-month mark reported an 8.8-point reduction in the PHQ-9 score. There did not appear to be an interactive effect between financial strain, the intervention, and depressive symptoms. The BTB intervention did not appear to reduce the effects of financial strain on depression; the authors noted that the magnitude of the reduction in depressive symptoms was greater among persons with low financial strain and, therefore, that efforts to reduce financial strain could improve the effectiveness of depression interventions.

#### The progressively lowered stress threshold (PLST) model and family meeting strategy for unpaid caregivers of persons with dementia

Robinson et al. (2016) studied a two-part intervention on the mental health of unpaid family caregivers for persons with dementia (PWD) [45]. The two-part intervention included the Progressively Lowered Stress Threshold (PLST) model and a family meeting strategy based on Mittelman’s New York University intervention [54]. The PLST model was an in-home psychoeducational curriculum about behavior management for family caregivers. Individual plans were created in response to caregiver needs at baseline. The family meeting strategy included at least one three-to-four-hour family education session focused on the use of community resources and on managing upsetting behavioral symptoms. Researchers reported a decrease in depressive symptoms during the 6-month survey, which was maintained over time (thus, they found a decrease in depressive symptoms from baseline to 6 months and then a stable prevalence of depressive symptoms at 6-, 12-, and 18-months). Among caregivers reporting financial strain, there was a significant drop in depressive symptoms over time, whereas, among caregivers reporting no financial strain, the depressive symptoms remained constant. The authors concluded that caregivers experiencing financial strain may benefit most from the intervention.

#### The protecting strong African American families (ProSAAF) for African American couples with children

Barton et al. (2018) studied the Protecting Strong African American Families (ProSAAF) intervention, which included six 2-hour sessions at participants’ homes in small towns and communities in Georgia. Sessions were led by trained facilitators from the region. Sessions focused on stressors such as racial discrimination and financial strain and provided African American couples with behavioral and cognitive techniques for handling stressors, including communication strategies between the couples. Children were invited to join the final 30 minutes of each session. Participants were surveyed at baseline, 9-month follow-up, and 17-month follow-up. Financial hardship was associated with reductions in relationship satisfaction, communication, confidence, and with increases in depressive symptoms (β = 0.82 [0.18], *p* < 0.07). There was an interactive effect of financial hardship with the intervention on relationship confidence but not depression. Thus, the authors found that the intervention modified the effect of financial strain on relationship confidence, but the intervention did not modify the effect of financial strain on depression.

Table 3 shows the characteristics of the interventions. While four of five interventions reduced depression, only two interventions (JOBS II and FRAME) appeared to reduce financial strain directly or indirectly and, in turn, reduce depression. Successful interventions had several characteristics in common: they were hosted over multiple sessions (rather than at one time), were led by trained facilitators, and were tailored to individual participants. All interventions highlighted in some ways the importance of social dynamics and contexts shaping health outcomes; for example, two studies included dyads and a third included a family session as part of the intervention. Effective interventions included coping mechanisms to improve one’s financial situation (i.e., finding employment), to modify cognitive behavior (i.e., reframing mindset), and to engage support (i.e., engaging social and community support). These findings highlight the importance of social networks and social support in shaping depression and its relation to stressors such as financial strain. Interventions were geared more toward reducing depression than addressing financial strain, pointing to a gap in the literature.

**Table 3:** Characteristics of interventions.

Intervention name	Population	Facilitator	Frequency of engagement	Educational components	Control group	Intervention effect on financial strain and depression
JOBS II intervention [52]	Recently unemployed job seekers	Two trainers (one man and one woman) led interactive workshops on enhancing job-search skills.	Five 4-hour sessions over the course of one week for groups of 12–22.	Teaching to anticipate setbacks and overcome barriers, build self-esteem, improve job-search efficacy, and increase locus of control.	Mailed a three-page pamphlet with information on the job-search process.	Financial strain mediated the relation between re-employment and reduced depressive symptoms.
The Fatherhood Relationship And Marriage Education (FRAME) intervention [53]	Low-income co-parenting couples with children between the ages of 1 and 18 years old	Program staff in partnership with community providers.	Families participated in the program 2 weeks following the program assignment, completing a baseline interview, 2-week post-intervention assessment, a 6-month assessment, and annual assessments	A “father-friendly, father-inclusive, family strengthening intervention” teaching (1) relationship education, (2) stress and coping skill training, and (3) child-centered parent training.	Randomized into intervention or control group by random-number generator; received an envelope with group assignment after completing the baseline assessment. Completed baseline and follow-up assessments.	Reductions in financial strain predicted decreased depressive symptoms in parents. The intervention improved primary and secondary coping for mothers and fathers, improving depressive symptoms. Reduced financial strain predicted improved father-child interactions.
Beat the Blues (BTB) [45]	African American older adults age 55 years or older with depressive symptoms (PHQ-9 score of 5 or greater).	Licensed social worker. In-home sessions were replaced by phone calls if there was “stable progress.”	Ten one-hour sessions weekly and then bi-weekly over a 4-month period.	Teaching education on depression, management of care, referral and linkage, stress reduction, and behavioral activation. Components were tailored to participant care needs, preferred techniques for stress reduction, and personal activity goals.	Put on the waitlist for intervention.	Participants in the intervention reported lower depressive symptoms scores after the 4-month intervention relative to the control group. There did not appear to be an interactive effect between financial strain, the intervention, and depressive symptoms.
The two-part intervention: the Progressively Lowered Stress Threshold (PLST) model and a family meeting strategy based on Mittelman’s New York University intervention. [70]	Unpaid caregivers of persons with dementia (PWD)	Facilitators created individualized caregiver intervention plans for each caregiver.	Support and assessment were ongoing for the 18 months of intervention. The family meeting strategy included at least one 3- to 4-h family education session	The PLST model is an in-home psychoeducational curriculum about behavior management for family caregivers. The family session focused on the use of community resources and on managing upsetting behavioral symptoms.	Caregiver indicators at baseline.	Caregivers with financial strain at baseline reported a significant decrease in depressive symptoms over time following the intervention.
The Protecting Strong African American Families (ProSAAF) [70]	African American couples with children	Sessions are led by trained facilitators.	Six 2-h sessions at participants’ homes.	Teaching behavioral and cognitive techniques for handling stressors. Additionally, sessions focused on communication techniques between the couples.	Given a book called “12 Hours to A Great Marriage” and a workbook.	Financial strain was associated with reductions in relationship satisfaction, communication, confidence, and with increases in depressive symptoms. Evidence of the interactive effect of financial strain with the intervention on relationship confidence but not depressive symptoms.

## Discussion

We aimed to scope the literature, to understand the relation between financial strain and depression, and to identify interventions to reduce depression. We found, first, a robust literature on financial strain and depression; while depression was consistently defined throughout the literature, financial strain was not. There were a substantial number of studies with female-only samples and none with male-only samples; multi-gender studies allow for comparison across genders, which can lead to a better understanding of the impact of financial strain on mental health across genders. Second, we found that financial strain was associated with depression across the literature. Eighty-eight percent of studies included found a significant, positive association between financial strain and depression. Thus, despite differences in the definition of financial strain, it was consistently positively associated with depression. Third, we identified five longitudinal articles that featured interventions to reduce depressive symptoms and included financial strain in their analyses. In four of the five studies, the intervention reduced depressive symptoms over time. It should be noted that these studies did not explicitly aim to reduce financial strain.

This article is consistent with the several articles that have reviewed constructs of financial strain and depression and contribute to a new synthesis of studies conducted within the United States. In their review of 40 global, observational studies quantifying the relation between financial strain and depression, Guan et al. found a positive association between measures of financial stress and depression in most studies, with stronger associations for populations with low income or low wealth [26]. Additionally, they found stronger associations between relative assets than absolute assets. Our study differs from Guan et al.’s in multiple ways; centrally, we included only longitudinal studies, and we focused on studies conducted in the United States, allowing for a more detailed synthesis of intervention designs. In a systematic review of different socioeconomic indicators, including financial strain and depression prognosis in randomized control trials, Buckman et al. found that struggling financially was related to worse depression prognosis; however, they found that controlling for employment attenuated the relationship and that there was no evidence of a significant association between financial strain and depression prognosis at 9–12 months [25]. Articles included in the review by Buckman et al. were all based in the United Kingdom, which has universal health coverage. Thus, the findings from these studies may not be directly relevant to the United States, where financial strain may have a stronger association with depression, given less generous social policies. In a study of the role of wealth and income in shaping depression in older adults across 16 European countries, Kourouklis et al. found that the relation between economic conditions and depression varied across regions and that neither wealth nor income was significantly associated with depression in Nordic countries, which have more generous social policies [55]. In this way, individual wealth and income may have had less of an effect on mental health because economic conditions—and financial needs that could result in financial strain if unmet—were being met through government or other societal structures. Thus, they concluded that the relation between economic precarity and depression may be stronger in countries that have less generous social policy.

This review is also consistent with previous reviews that have assessed the social and economic factors that shape mental health. As early as 1980, Dooley and Catalano documented the literature showing that negative economic change was associated with subsequent increases in behavioral disorders [56]. Muntaner et al. reviewed studies between 1999 and 2003 that used language around “social class” or “socioeconomic status” and major psychiatric disorders; they found that reduced socioeconomic position was associated with major mental disorders [4]. Pollack et al. reviewed studies from 1990 to 2006 assessing the relation between wealth and health outcomes more broadly. They identified six studies at the time that examined the relation between mental health and wealth, finding that five of the six studies identified a significant relation between low wealth and mental health in subsamples or the entire samples studied [6]. Osypuk et al. conducted a review of social and economic policies and their effect on health. They focused on four policy domains—housing, employment, marriage/family strengthening, and income support—across several health factors, including mental health. They identified 15 studies that measured mental health indicators following policy implementation and reported that 8 of these studies documented a health benefit of the policy [5]. Mair et al. reviewed the literature on neighborhood factors and mental health, identifying 45 articles published from 1990 to 2007. They found that more articles featured the association between depression and social processes (such as social interactions, violence, and disorder) than structural features (such as built environment and socioeconomic and racial composition of neighborhoods) and called for more literature to assess the causal effects of area characteristics on depression [57]. Wetherall et al. reviewed studies from inception to 2017 on social rank and depression; they synthesized 70 articles by the following measurements: social comparison scale (*n* = 32), subjective social status (*n* = 32), and other indicators of social rank (*n* = 6). They concluded that the majority of studies found that lower social rank was associated with more depressive symptoms [58]. They called for more research to better understand how one’s perception of social position compared to others relates to depression. Thus, the current review is consistent with other reviews on social and economic factors in shaping mental health but adds clarity to the literature on the perception of financial standing—i.e., financial strain—and depression in particular.

We identified a gap in the literature on the relation between financial strain and depression among racial and ethnic minoritized populations and migrant and refugee populations in particular, which have recently been highlighted by international bodies as an important population to consider [59, 60]. We found that Asian populations were the least represented racial group in this review. In the United States, the relation between assets and depression among racial and ethnic minoritized populations is complex [61], with non-US-born persons reporting better mental health than US-born counterparts and with paradoxical findings on depression emerging that may be in part explained by unequal access to assets [10]. Additionally, work outside of the United States has shown the associations between economic stressors and mental health among minoritized populations [62] through differential access to healthcare and employment opportunities, despite interventions to support the mental health of minoritized groups [63, 64]. Populations who migrate are a diverse group [60] with different financial situations; expanding research on this group could help illuminate the different experiences that this group has and how they may shape mental health more broadly. Given heightened exposure to stressors such as financial strain in racial and ethnic minoritized populations and among many populations who migrate, future work focusing on interventions to reduce economic inequities and improve depression among these populations could potentially improve mental health in these groups.

Of the fifty-eight longitudinal studies identified, only five featured interventions to reduce depression, and only two aimed to measure a reduction in financial strain from the intervention (as opposed to measuring differences in the efficacy of interventions on reducing depression by financial strain status). One of these two studies implemented an intervention to help recently unemployed job seekers gain re-employment [48], while the other study implemented an intervention to help parents cope with stressors such as financial strain, including primary coping (i.e., problem-solving to regain employment) and secondary coping (i.e., cognitive restructuring and engaging social support) [52]. The intervention studies highlighted several features of the relation between financial strain and depression. For example, they drew attention to the importance of social support, which moderated the relation between financial strain and depression in many of the non-intervention studies. Interventions were tailored to individuals, and families were included, recognizing the role of context in shaping depression. Given the limited literature on men in particular, the FRAME intervention provides an important focus on the relation among financial strain, depression, and broader family context among men and the potential benefits of coping interventions [52]. However, the overall limited number of interventions focused on reducing financial strain, which is clearly associated with depression, speaks to a larger gap in the literature and calls for research in the future that includes interventions to address and reduce financial strain. In a review specifically conducted on reducing the impact of financial hardship on depression, Moore et al. find that ‘job club’ programs targeted at unemployed persons may be effective, although more current and varied data are needed [65].

This review should be considered in light of two limitations. Because we selected articles that reported the relation between depression and financial strain, it is possible that the articles selected may embed publication bias. That is, studies that controlled for financial strain and did not find a significant relation between depression and financial strain may have been less likely to be in the published literature and, therefore, may be excluded from our analyses. Therefore, the studies represented in this review may reflect a stronger relation between financial strain and depression; however, no literature to date has shown that financial strain is associated with less depression, suggesting that the association remains an important factor in the context that shapes health outcomes. Second, it is possible that there is work on related and contributory constructs that do not use the terms captured in our search strategy and were not included in our review. We aimed to do a comprehensive search of the literature across disciplines, but it is possible that there are other works that assess the role of subjective relations to money and related concepts that were not captured in this review.

Notwithstanding these limitations, these findings suggest that the association between financial strain and depression over time has been well established. While the definition of financial strain varies, the patterns that have emerged are clear: having more financial strain is associated with poor mental health. The literature has gaps that should be addressed, such as an under-representation of Asian populations in studies conducted in the United States and of other minoritized ethnic groups in many studies worldwide. The literature also lacks standard definitions and measurement approaches to financial strain, making it challenging to compare findings across studies, that merit further attention. Additionally, there is a paucity of studies on interventions in the United States that may reduce the ill effects of financial strain on depression, and much work can be done to identify ways to reduce financial strain (and prevent depression) and to mitigate its consequences. While addressing the root causes of financial strain are costly, the costs of not addressing depression continue to rise. It is costly not to invest in the prevention of mental illness [66]. Estimates suggest that depression cost the US $326 billion in 2018, with a rising share being shouldered by workplaces [67]. With elevated levels of depression reported during the COVID-19 pandemic [68] and adolescents aging into adulthood, the burden of poor mental health is likely to rise, along with costs to individuals, employers, and families. Addressing financial strain, particularly as economies grapple with inflation and adjust to a post-COVID-19 world, may help to reduce the burden of depression. Additionally, coping mechanisms suggested in the interventions may provide benefits for a host of life stressors beyond financial strain. Interventions to improve financial strain and depression could be valuable in multiple ways, given the potential multidirectional relations between financial strain and recurrent depression—and recurrent depression on financial standing. Therefore, intervening at different levels may be a cost-effective, politically appealing solution and may lead to improved mental health in the face of multiple stressors [53].

## Conclusion

Financial strain is a formidable factor associated with depression in populations. The association between financial strain and depression is consistent, positive, and significant in the United States. The divergent economic outcomes following the COVID-19 pandemic [27] may contribute to greater financial strain and divergent mental health outcomes across populations [69]. As the economic effects of the COVID-19 pandemic are felt, attention should be paid to financial strain and its attendant consequences on population’s mental health, with possibilities of increased mental health burden in the months and years to come.

## Supplementary information

Supplemental Materials.pdf

## References

[1] Vahratian A (2021) Symptoms of anxiety or depressive disorder and use of mental health care among adults during the COVID-19 pandemic — United States, August 2020–February 2021. MMWR Morb Mortal Wkly Rep. 2021;70:490–94. 10.15585/mmwr.mm7013e2 PMC8022876 33793459

[2] Kessler RC (2012). The costs of depression. Psychiatr Clin North Am.

[3] Galea S, Ettman CK (2021). Mental health and mortality in a time of COVID-19. Am J Public Health.

[4] Muntaner C (2004). Socioeconomic position and major mental disorders. Epidemiol Rev.

[5] Osypuk TL, Joshi P, Geronimo K, Acevedo-Garcia D (2014). Do social and economic policies influence health? A review. Curr Epidemiol Rep.

[6] Pollack CE, Chideya S, Cubbin C, Williams B, Dekker M, Braveman P (2007). Should health studies measure wealth?: a systematic review. Am J Preventive Med.

[7] Allen J, Balfour R, Bell R, Marmot M (2014). Social determinants of mental health. Int Rev Psychiatry.

[8] Allen J, Marmot M, World Health Organization, Fundação Calouste Gulbenkian (2014) Social determinants of mental health.

[9] Ettman CK, Cohen GH, Galea S (2020). Is wealth associated with depressive symptoms in the United States. Ann Epidemiol.

[10] Ettman CK, Cohen GH, Abdalla SM, Galea S (2020). Do assets explain the relation between race/ethnicity and probable depression in U.S. adults?. PLoS ONE.

[11] Ettman CK, Cohen GH, Vivier PM, Galea S. Savings, home ownership, and depression in low-income US adults. Soc Psychiatry Psychiatr Epidemiol. 2020;56:1211–9. 10.1007/s00127-020-01973-y PMC8110606 33175205

[12] Patel V, Burns JK, Dhingra M, Tarver L, Kohrt BA, Lund C (2018). Income inequality and depression: a systematic review and meta-analysis of the association and a scoping review of mechanisms. World Psychiatry.

[13] Dijkstra-Kersten SMA, Biesheuvel-Leliefeld KEM, van der Wouden JC, Penninx BWJH, van Marwik HWJ (2015). Associations of financial strain and income with depressive and anxiety disorders. J Epidemiol Community Health.

[14] McGovern P, Nazroo JY (2015). Patterns and causes of health inequalities in later life: a Bourdieusian approach. Sociol Health Illn.

[15] Wilkinson LR (2016). Financial strain and mental health among older adults during the Great Recession. GERONB.

[16] Festinger L (1954). A theory of social comparison processes. Hum Relat.

[17] Mirowsky J, Ross CE (1999). Economic hardship across the life course. Am Sociol Rev.

[18] Stansfeld S, Candy B (2006). Psychosocial work environment and mental health—a meta-analytic review. Scand J Work Environ Health.

[19] Monroe SM, Harkness KL (2005). Life stress, the “Kindling” Hypothesis, and the recurrence of depression: considerations from a life stress perspective. Psychol Rev.

[20] Monroe SM, Anderson SF, Harkness KL (2019). Life stress and major depression: the mysteries of recurrences. Psychol Rev.

[21] Kendler KS, Karkowski LM, Prescott CA (1999). Causal relationship between stressful life events and the onset of major depression. AJP.

[22] Brown GW, Harris TO, Kendrick T, Chatwin J, Craig TKJ, Kelly V (2010). Antidepressants, social adversity and outcome of depression in general practice. J Affect Disord.

[23] Monroe SM, Torres LD, Guillaumot J, Harkness KL, Roberts JE, Frank E (2006). Life stress and the long-term treatment course of recurrent depression: III. Nonsevere life events predict recurrence for medicated patients over 3 years. J Consult Clin Psychol.

[24] Solomon DA, Keller MB, Leon AC, Mueller TI, Lavori PW, Shea MT (2000). Multiple recurrences of major depressive disorder. Am J Psychiatry.

[25] Buckman JEJ, Saunders R, Stott J, Cohen ZD, Arundell L-L, Eley TC (2022). Socioeconomic indicators of treatment prognosis for adults with depression: a systematic review and individual patient data meta-analysis. JAMA Psychiatry.

[26] Guan N, Guariglia A, Moore P, Xu F, Al-Janabi H (2022). Financial stress and depression in adults: a systematic review. PLoS ONE.

[27] Long H, van Dam A, Fowers A, Shapiro L (2020) The covid-19 recession is the most unequal in modern U.S. history. The Washington Post.

[28] Hertz-Palmor N, Moore TM, Gothelf D, DiDomenico GE, Dekel I, Greenberg DM (2021). Association among income loss, financial strain and depressive symptoms during COVID-19: evidence from two longitudinal studies. J Affect Disord.

[29] Ettman CK, Abdalla SM, Cohen GH, Sampson L, Vivier PM, Galea S (2021). Low assets and financial stressors associated with higher depression during COVID-19 in a nationally representative sample of US adults. J Epidemiol Community Health.

[30] Tricco AC, Lillie E, Zarin W, O’Brien KK, Colquhoun H, Levac D (2018). PRISMA extension for scoping reviews (PRISMA-ScR): checklist and explanation. Ann Intern Med.

[31] Rathbone J, Hoffmann T, Glasziou P (2015). Faster title and abstract screening? Evaluating Abstrackr, a semi-automated online screening program for systematic reviewers. Syst Rev.

[32] Trikalinos T, Wallace B, Jap J, Senturk B, Adam G, Smith B (2020). 41st annual meeting of the society for medical decision making, Portland, Oregon, October 20–23, 2019. Med Decis Mak.

[33] Peters MDJ, Godfrey CM, Khalil H, McInerney P, Parker D, Soares CB (2015). Guidance for conducting systematic scoping reviews. Int J Evid-Based Healthc.

[34] Peters MDJ, Godfrey C, McInerney P, Munn Z, Tricco AC, Khalil, H. Chapter 11: Scoping Reviews (2020 version). In: Aromataris E, Munn Z, Editors. JBI manual for evidence synthesis. Joanna Briggs Institute; 2020. Available from https://synthesismanual.jbi.global. 10.46658/JBIMES-20-12..

[35] Wang D, Li Y-L, Qiu D, Xiao S-Y (2021). Factors influencing paternal postpartum depression: a systematic review and meta-analysis. J Affect Disord.

[36] Dohrenwend BS, Askenasy AR, Krasnoff L, Dohrenwend BP (1978). Exemplification of a method for scaling life events: the PERI life events scale. J Health Soc Behav.

[37] Pearlin LI, Menaghan EG, Lieberman MA, Mullan JT (1981). The stress process. J Health Soc Behav.

[38] Conger RD, Ge X, Elder GH, Lorenz FO, Simons RL (1994). Economic stress, coercive family process, and developmental problems of adolescents. Child Dev.

[39] Law EF, Zhou C, Seung F, Perry F, Palermo TM (2021). Longitudinal study of early adaptation to the coronavirus disease pandemic among youth with chronic pain and their parents: effects of direct exposures and economic stress. Pain.

[40] Mccormick N, Trupin L, Yelin EH, Katz PP (2018). Socioeconomic predictors of incident depression in systemic lupus erythematosus. Arthritis Care Res.

[41] Gutierrez IA, Park CL, Wright BRE (2017). When the divine defaults: religious struggle mediates the impact of financial stressors on psychological distress. Psychol Relig Spiritual.

[42] Yoshikawa H, Godfrey EB, Rivera AC (2008). Access to institutional resources as a measure of social exclusion: Relations with family process and cognitive development in the context of immigration. N. Directions Child Adolesc Dev.

[43] Mendes De Leon CF, Rapp SS, Kasl SV (1994). Financial strain and symptoms of depression in a community sample of elderly men and wWomen: a longitudinal study. J Aging Health.

[44] Valentino SW, Moore JE, Cleveland MJ, Greenberg MT, Tan X (2014). Profiles of financial stress over time using subgroup analysis. J Fam Econ Iss.

[45] Robinson KM, Crawford TN, Buckwalter K (2016). Outcomes of a two-component, evidence- based intervention on depression in dementia caregivers. Best Pract Ment Health.

[46] Curran MA, Li X, Barnett M, Kopystynska O, Chandler AB, LeBaron AB (2021). Finances, depressive symptoms, destructive conflict, and coparenting among lower-income, unmarried couples: a two-wave, cross-lagged analysis. J Fam Psychol.

[47] Jones SMW, Nguyen T, Chennupati S (2020). Association of financial burden with self-rated and mental health in older adults with cancer. J Aging Health.

[48] Vinokur AD, Schul Y (1997). Mastery and inoculation against setbacks as active ingredients in the JOBS intervention for the unemployed. J Consult Clin Psychol.

[49] Vinokur AD, Price RH, Caplan RD (1996). Hard times and hurtful partners: how financial strain affects depression and relationship satisfaction of unemployed persons and their spouses. J Personal Soc Psychol.

[50] Vinokur AD, Schul Y (2002). The web of coping resources and pathways to reemployment following a job loss. J Occup Health Psychol.

[51] Price RH, Choi JN, Vinokur AD (2002). Links in the chain of adversity following job loss: how financial strain and loss of personal control lead to depression, impaired functioning, and poor health. J Occup Health Psychol.

[52] Wadsworth Martha E, Rindlaub L, Hurwich-Reiss E, Rienks S, Bianco H, Markman HJ (2013). A longitudinal examination of the adaptation to poverty-related stress model: predicting child and adolescent adjustment over time. J Clin Child Adolesc Psychol.

[53] Szanton SL, Thorpe RJ, Gitlin LN (2014). Beat the blues decreases depression in financially strained older African-American adults. Am J Geriatr Psychiatry.

[54] Mittelman MS, Ferris SH, Shulman E, Steinberg G, Ambinder A, Mackell JA (1995). A comprehensive sSupport program: effect on depression in spouse-caregivers of AD patients1. Gerontologist.

[55] Kourouklis D, Verropoulou G, Tsimbos C (2019) The impact of wealth and income on the depression of older adults across European welfare regimes. Ageing Soc. 2019;40:2448–79.

[56] Dooley D, Catalano R (1980). Economic change as a cause of behavioral disorder. Psychol Bull.

[57] Mair C, Diez Roux AV, Galea S (2008). Are neighbourhood characteristics associated with depressive symptoms? A review of evidence. J Epidemiol Community Health.

[58] Wetherall K, Robb KA, O’Connor RC (2019). Social rank theory of depression: a systematic review of self-perceptions of social rank and their relationship with depressive symptoms and suicide risk. J Affect Disord.

[59] World Health Organization. World report on the health of refugees and migrant. Geneva: World Health Organization; 2022.

[60] Galea S, Ettman CK, Zaman M. Migration and health. Chicago: University of Chicago Press; 2022.

[61] Ettman CK, Koya SF, Fan A, Robbins G, Shain J, Cozier Y, et al. More, less, or the same: a scoping review of studies that compare depression between Black and White U.S. adult populations. SSM - Mental Health. 2022;2:100161. 10.1016/j.ssmmh.2022.100161.

[62] Barnett P, Mackay E, Matthews H, Gate R, Greenwood H, Ariyo K (2019). Ethnic variations in compulsory detention under the Mental Health Act: a systematic review and meta-analysis of international data. Lancet Psychiatry.

[63] Memon A, Taylor K, Mohebati LM, Sundin J, Cooper M, Scanlon T (2016). Perceived barriers to accessing mental health services among black and minority ethnic (BME) communities: a qualitative study in Southeast England. BMJ Open.

[64] Arundell L-L, Barnett P, Buckman JEJ, Saunders R, Pilling S (2021). The effectiveness of adapted psychological interventions for people from ethnic minority groups: a systematic review and conceptual typology. Clin Psychol Rev.

[65] Moore THM, Kapur N, Hawton K, Richards A, Metcalfe C, Gunnell D (2017). Interventions to reduce the impact of unemployment and economic hardship on mental health in the general population: a systematic review. Psychol Med.

[66] McDaid D, Park A-L, Wahlbeck K (2019). The economic case for the prevention of mental illness. Annu Rev Public Health.

[67] Greenberg PE, Fournier A-A, Sisitsky T, Simes M, Berman R, Koenigsberg SH (2021). The economic burden of adults with major depressive disorder in the United States (2010 and 2018). PharmacoEconomics.

[68] Ettman CK, Fan AY, Subramanian M, Adam GP, Badillo Goicoechea E, Abdalla SM (2023). Prevalence of depressive symptoms in U.S. adults during the COVID-19 pandemic: a systematic review. SSM - Popul Health.

[69] Ettman CK, Cohen GH, Abdalla SM, Sampson L, Trinquart L, Castrucci BC (2022). Persistent depressive symptoms during COVID-19: a national, population-representative, longitudinal study of U.S. adults. Lancet Reg Health Am.

[70] Barton AW, Beach SRH, Bryant CM, Lavner JA, Brody GH (2018). Stress spillover, African Americans’ couple and health outcomes, and the stress-buffering effect of family-centered prevention. J Fam Psychol.

