# Supplementary material for: Financial strain and depression in the U.S.: a scoping review
Source: Transl Psychiatry. 2023 May 13;13:168. doi: 10.1038/s41398-023-02460-z (PMC10182750; doi:10.1038/s41398-023-02460-z)
Supplement: Supplementary file 1 — Supplemental Materials.pdf [file 41398_2023_2460_MOESM1_ESM.pdf]

Appendix A. Search Strategy

Searches conducted through January 19, 2023

Pubmed search:

("Depressive Disorder"[Mesh] OR Depression[tw] OR "Psychological Distress"[Mesh] OR psychological distress[tw] OR depress*[tw] OR Common mood disorder[tw]) AND ("Economic Status"[Mesh] OR wealth[tw] OR savings[tw] OR financial strain[tw] OR debt[tw])

Filters applied: humans; English language

Ebsco search, in Medline, PsycINFO, PsycArticles, SocINDEX, and EconLit:

(Depression OR depressive OR psychological distress OR depress* OR Common mood disorder) AND (wealth OR financial strain OR savings OR debt)

Select: Scholarly (Peer Reviewed) Journals; English language; human subject

Embase search:

#1 'depression'/exp OR depression
#2 depression OR depressive OR 'psychological distress' OR depress* OR 'common mood disorder' OR 'psychiatric diagnosis'
#3 #1 OR #2
#4 wealth OR 'financial strain' OR savings OR debt
#5 #3 AND #4
#6 #3 AND #4 AND ([article]/lim OR [article in press]/lim) AND [english]/lim

Combined

Appendix B. Articles included in review (n=58)

Adesogan, O., Lavner, J. A., Carter, S. E., & Beach, S. R. H. (2022). Covid-19 stress and the health of black americans in the rural south. Clinical Psychological Science

Angel, R. J., Frisco, M., Angel, J. L., Chiriboga, D. A. (2003) Financial strain and health among elderly Mexican-origin individuals. J Health Soc Behav

Barton, Allen W., Beach, Steven R. H., Bryant, Chalandra M., Lavner, Justin A., Brody, Gene H. (2018) Stress spillover, African Americans,Äô couple and health outcomes, and the stress-buffering effect of family-centered prevention. Journal of Family Psychology

Bialowolski, P., Weziak-Bialowolska, D., Lee, M. T., Chen, Y., VanderWeele, T. J., & McNeely, E. (2021). The role of financial conditions for physical and mental health. Evidence from a longitudinal survey and insurance claims data. Social Science & Medicine

Buckingham-Howes, S., Holmes, K., Glenn Morris, J., Grattan, Lynn M. (2018) Prolonged financial distress after the Deepwater Horizon oil spill predicts behavioral health. The Journal of Behavioral Health Services & Research

Cao, H., Zhou, N., Li, X., Serido, J., & Shim, S. (2021). Temporal dynamics of the association between financial stress and depressive symptoms throughout the emerging adulthood. Journal of Affective Disorders

Cohen, C. I., Goh, K. H., Gustave, M. (2010) A prospective study of outcome and predictors of subclinical and clinical depression in an older biracial sample of psychiatric outpatients. J Affect Disord

Curran, M. A., Li, X., Barnett, M., Kopystynska, O., Chandler, A. B., & LeBaron, A. B. (2021). Finances, depressive symptoms, destructive conflict, and coparenting among lower-income, unmarried couples: A two-wave, cross-lagged analysis. Journal of Family Psychology

Forbes, M. K. Krueger, R. F. (2019) The Great Recession and mental health in the United States. Clinical Psychological Science

Galatzer-Levy, I. R., Bonanno, G. A. (2012) Beyond normality in the study of bereavement: Heterogeneity in depression outcomes following loss in older adults. Social Science & Medicine

Galtieri, L. R., Fladeboe, K. M., King, K., Friedman, D., Compas, B., Breiger, D., Lengua, L., Keim, M., Boparai, S., & Katz, L. F. (2022). Caregiver perceived financial strain during pediatric cancer treatment: Longitudinal predictors and outcomes. Health Psychology

Gilman, S. E., Bruce, M. L., Ten Have, T., Alexopoulos, G. S., Mulsant, B. H., Reynolds, C. F., 3rd, Cohen, A. (2013) Social inequalities in depression and suicidal ideation among older primary care patients. Soc Psychiatry Psychiatr Epidemiol

Gutierrez, I.A. Park, C.L., Wright, B. R. E. (2017) When the divine defaults: Religious struggle mediates the impact of financial stressors on psychological distress. Psychology of Religion and Spirituality

Hertz-Palmor, N., Moore, T. M., Gothelf, D., DiDomenico, G. E., Dekel, I., Greenberg, D. M., Brown, L. A., Matalon, N., Visoki, E., White, L. K., Himes, M. M., Schwartz-Lifshitz, M., Gross, R., Gur, R. C., Gur, R. E., Pessach, I. M., & Barzilay, R.

(2021). Association among income loss, financial strain and depressive symptoms during COVID-19: Evidence from two longitudinal studies. Journal of Affective Disorders

Horwitz, A. V., McLaughlin, J., White, H. R. (1998) How the negative and positive aspects of partner relationships affect the mental health of young married people. Journal of Health and Social Behavior

Horwitz, S. M., Briggs-Gowan, M. J. Storfer-Isser, A. Carter, A. S. (2007) Prevalence, correlates, and persistence of maternal depression. J Womens Health (Larchmt)

Jackson, A. P., Bender, P. M., Franke, T. M. (2008) Low-wage maternal employment and parenting style. Social Work

Johnson, A. K., Fulco, C. J., Augustyn, M. B. (2019) Intergenerational continuity in alcohol misuse: Maternal alcohol use disorder and the sequelae of maternal and family functioning.Psychol Addict Behav

Jones, S. M. W., Nguyen, T., & Chennupati, S. (2020). Association of financial burden with self-rated and mental health in older adults with cancer. Journal of Aging and Health

Kasen, S., Cohen, P., Chen, H., Must, A. (2008) Obesity and psychopathology in women: a three decade prospective study. Int J Obes (Lond)

Krause, N. (1987) Chronic strain, locus of control, and distress in older adults. Psychol Aging

Krause, N. and Thompson E (1998). Cognitive functioning, stress, and psychological well-being in later life. Journal of Mental Health and Aging

Krause, N. (2009) Religious involvement, gratitude, and change in depressive symptoms over time. International Journal for the Psychology of Religion

Law, E. F., Zhou, C., Seung, F., Perry, F., & Palermo, T. M. (2021). Longitudinal study of early adaptation to the coronavirus disease pandemic among youth with chronic pain and their parents: Effects of direct exposures and economic stress. Pain

Leonard I. Pearlin, Elizabeth G. Menaghan, Morton A. Lieberman and Joseph T. Mullan (1981) The stress process. Journal of Health and Social Behavior

Lipton, R. (1994) The effect of moderate alcohol use on the relationship between stress and depression. American Journal of Public Health

McCormick, N., Trupin, L., Yelin, E. H., Katz, P. P. (2018) Socioeconomic Predictors of Incident Depression in Systemic Lupus Erythematosus. Arthritis Care Res (Hoboken)

Mendes de Leon, C. F., Rapp, S. S., Kasl, S. V. (1994) Financial strain and symptoms of depression in a community sample of elderly men and women: A longitudinal study. Journal of Aging and Health

Mitchell, A. M., Christian, L. M. (2017) Financial strain and birth weight: the mediating role of psychological distress. Arch Womens Ment Health

Monserud, M. A. (2019) Marital Status and Trajectories of Depressive Symptoms Among Older Adults of Mexican Descent. Int J Aging Hum Dev

Monserud, M. A., Markides, K. S. (2017) Changes in depressive symptoms during widowhood among older Mexican Americans: the role of financial strain, social support, and church attendance. Aging Ment Health

Nam, I. (2016) Financial difficulty effects on depressive symptoms among dementia patient caregivers. Community Mental Health Journal
Neppl, T. K., Senia, J. M., Donnellan, M. B. (2016) Effects of economic hardship: Testing the family stress model over time. Journal of Family Psychology
Newland, R. P., Crnic, K. A., Cox, M. J. Mills-Koonce, W. R. (2013) The family model stress and maternal psychological symptoms: Mediated pathways from economic hardship to parenting. Journal of Family Psychology
Nishtala, M. V., Robbins, S. E., Savage, S., Timsina, L. R., Murphy, P. B., Marka, N. A., Venkatesh, M., & Zarzau, B. L. (2022). Patients follow different financial hardship trajectories in the year after injury. Annals of Surgery
O'Neal, C W., Arnold, A.L., Lucier-Greer, M., Wickrama, K. A. S., Bryant, C. M. (2015) Economic pressure and health and weight management behaviors in African American couples: A family stress perspective. Journal of Health Psychology
Price, R. H., Choi, J. N., Vinokur, A. D. (2002) Links in the chain of adversity following job loss: how financial strain and loss of personal control lead to depression, impaired functioning, and poor health. J Occup Health Psychol
Reisinger, E. L., DiIorio, C. (2009) Individual, seizure-related, and psychosocial predictors of depressive symptoms among people with epilepsy over six months. Epilepsy Behav
Robinson, K.M., Crawford, T. N., Buckwalter, K. (2016) Outcomes of a two-component, evidence-based intervention on depression in dementia caregivers. Best Practices in Mental Health: An International Journal
Russell, D. W., Clavél,, F. D. Cutrona, C. E., Abraham, W. T., Burzette, R. G. (2018) Neighborhood racial discrimination and the development of major depression. J Abnorm Psychol
Saasa, S., Ward, K. P., Sandberg, S., & Jacobson, J. (2021). Financial hardship, neighborhood cohesion and child externalizing behaviors: An extension of the family stress model among immigrant mothers. Children and Youth Services Review
Seto, M., Cornelius, M. D., Goldschmidt, L., Morimoto, K., Day, N. L. (2005) Long-term Effects of Chronic Depressive Symptoms Among Low-Income Childrearing Mothers. Matern Child Health J
Shelleby, E. C., Pittman, L. D., Bridgett, D. J., Keane, J., Zolinski, S., & Caradec, J. (2022). Associations between local COVID-19 case rates, pandemic-related financial stress and parent and child functioning. Journal of Family Psychology
Shippee, T. P., Wilkinson, L. R., Schafer, M. H., Shippee, N. D. (2019) Long-Term Effects of Age Discrimination on Mental Health: The Role of Perceived Financial Strain. J Gerontol B Psychol Sci Soc Sci
Strawbridge, W. J., Deleger, S., Roberts, R. E., Kaplan, G. A. (2002) Physical activity reduces the risk of subsequent depression for older adults. Am J Epidemiol
Szanton, S. L., Thorpe Jr, R. J., Gitlin, L. N. (2014) Beat the Blues decreases depression in financially strained older African-American adults. American Journal of Geriatric Psychiatry
Uebelacker, L. A., Eaton, C. B., Weisberg, R., Sands, M., Williams, C., Calhoun, D., Manson, J. E., Denburg, N. L., Taylor, T. (2013) Social support and physical activity as moderators of life stress in predicting baseline depression and change

in depression over time in the Women's Health Initiative. Social Psychiatry and Psychiatric Epidemiology: The International Journal for Research in Social and Genetic Epidemiology and Mental Health Services
Valentino, S. W., Moore, J. E., Cleveland, M. J., Greenberg, M. T.,Tan, X. (2014) Profiles of financial stress over time using subgroup analysis. Journal of Family and Economic Issues
Vinokur, A. D., Price, R. H., Caplan, R. D. (1996) Hard times and hurtful partners: how financial strain affects depression and relationship satisfaction of unemployed persons and their spouses. J Pers Soc Psychol
Vinokur, A. D., Schul, Y. (2002) The web of coping resources and pathways to reemployment following a job loss. J Occup Health Psychol
Vinokur, A. D., Schul, Y. (1997) Mastery and inoculation against setbacks as active ingredients in the JOBS intervention for the unemployed. J Consult Clin Psychol
Wadsworth, M. E., Rindlaub, L., Hurwich-Reiss, E., Rienks, S., Bianco, H., Markman, H. J. (2013) A longitudinal examination of the adaptation to poverty-related stress model: Predicting child and adolescent adjustment over time. Journal of Clinical Child and Adolescent Psychology
West, K. B., Hale, M. E., Roche, K. M., White, R. M. B., & Suveg, C. (2022). Predictors of latent class trajectories of depressive symptoms in Latina mothers. Journal of Family Psychology
Wickrama, K. A. S., Klopack, E. T., & O’Neal, C. W. (2022). Higher-order trajectories of pain and depressive symptoms link midlife financial stress to women’s well-being in later life. Aging & Mental Health
Wickrama, K. A. S., & O’Neal, C. W. (2021). Midlife marital and financial stress and the progression of later-life health problems for husbands and wives. Journal of Aging and Health
Wickrama, K. A. S., O'Neal, C.W., Lorenz, F. O. (2018) Marital processes linking economic hardship to mental health: The role of neurotic vulnerability. Journal of Family Psychology
Wilkinson, L. R. (2016) Financial Strain and Mental Health Among Older Adults During the Great Recession.J Gerontol B Psychol Sci Soc Sci
Yoshikawa, H., Godfrey, E. B., Rivera, A. C. (2008) Access to institutional resources as a measure of social exclusion: relations with family process and cognitive development in the context of immigration. New Dir Child Adolesc Dev
